# PSpice Modeling of a Sandwich Piezoelectric Ceramic Ultrasonic Transducer in Longitudinal Vibration

**DOI:** 10.3390/s17102253

**Published:** 2017-09-30

**Authors:** Xiaoyuan Wei, Yuan Yang, Wenqing Yao, Lei Zhang

**Affiliations:** Department of Electronic Engineering, Xi’an University of Technology, Xi’an 710048, Shaanxi, China; wxy@stu.xaut.edu.cn (X.W.); yaowenqing@stu.xaut.edu.cn (W.Y.); leizhang830102@stu.xaut.edu.cn (L.Z.)

**Keywords:** PSpice model, sandwiched piezoelectric ultrasonic transducer, longitudinal vibration, impedance analysis, transient analysis

## Abstract

Sandwiched piezoelectric transducers are widely used, especially in high power applications. For more convenient analysis and design, a PSpice lossy model of sandwiched piezoelectric ultrasonic transducers in longitudinal vibration is proposed by means of the one-dimensional wave and transmission line theories. With the proposed model, the resonance and antiresonance frequencies are obtained, and it is shown that the simulations and measurements have good consistency. For the purpose of further verification the accuracy and application of the PSpice model, a pitch-catch setup and an experimental platform are built. They include two sandwiched piezoelectric ultrasonic transducers and two aluminum cylinders whose lengths are 20 mm and 100 mm respectively. Based on this pitch-catch setup, the impedance and transient analysis are performed. Compared with the measured results, it is shown that the simulated results have good consistency. In addition, the conclusion can be drawn that the optimal excitation frequency for the pitch-catch setup is not necessarily the resonance frequency of ultrasonic transducers, because the resonance frequency is obtained under no load. The proposed PSpice model of the sandwiched piezoelectric transducer is more conveniently applied to combine with other circuits such as driving circuits, filters, amplifiers, and so on.

## 1. Introduction

**Figure 1:**
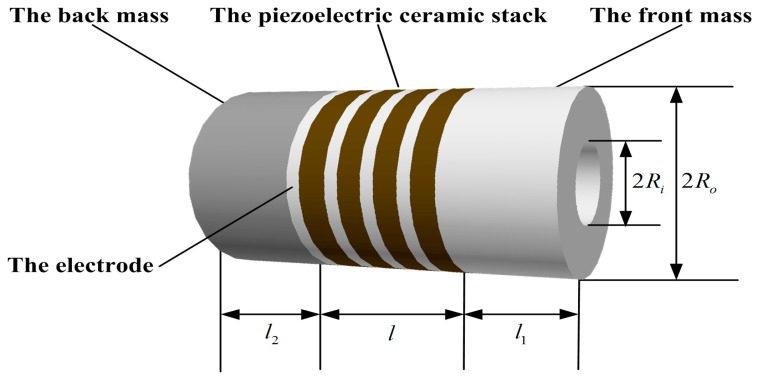
Simplified structure diagram of a sandwich piezoelectric ultrasonic transducer.

The sandwiched piezoelectric ultrasonic transducer vibrating in longitudinal mode is also called the Langevin composite transducer. It consists of metal front and back masses, piezoelectric ceramic stack, metal electrodes and the prestressed bolt. Based on the conventional design theory about the sandwiched piezoelectric transducers vibrating in longitudinal mode as shown in Figure 1, it is required that the diameter be smaller than its longitudinal dimension, in order that the one-dimensional longitudinal vibration theory be applied [1]. It is regarded as a fundamental component in various ultrasonic applications [1,2,3], and is widely used in high power ultrasonic fields including ultrasonic detecting, ultrasonic welding, underwater sound communication, and so on. Moreover, it has some advantages such as high-power capacity, high electro-acoustic conversion efficiency, low losses in mechanical and dielectric, and so on.

It is well known that the theory analysis for piezoelectric components is a vital basis for the design of piezoelectric transducers. So far there are a few methods used in analyzing piezoelectric transducers. Among the methods, the equivalent circuit method is widely used, because it is brief and clear in its physical meaning. In addition, mechanical and dielectric losses can be easily taken into account using the equivalent circuit method. The piezoelectric ceramic disks or rings vibrating in the thickness direction have been successfully modeled by using equivalent circuit methods which mainly include Mason’s [4], Redwood’s [5] and Krimholtz Leedom Matthaei (KLM) models [6]. In order to achieve these equivalent circuits mentioned above on the circuit analysis softwares such as PSpice and Spice, many efforts have been made [7,8]. In view of the circuit models mentioned above containing negative capacitance and transformer, based on the controlled sources and the lossless transmission line, an elegant equivalent circuit model for piezoelectric ceramic disks or rings vibrating in the thickness direction is proposed by Leach [9]. Then the simulation program with integrated circuit emphasis (SPICE) equivalent circuit model was proposed by Puttmer A. [10] to investigate the effect of the losses in mechanical and dielectric. Van D.J. et al. [11] used the approach of Puttmer A. et al. [10] to investigate the speed of acoustic and attenuation in solids and liquids. Guisado A. et al. [12] applied the Leach’s model to obtain the most accurate equivalent circuit of piezoceramic vibrating in thickness mode by using PSpice. The above published works aimed at obtaining some material acoustic parameters and the accurate equivalent circuit based on a single piezoelectric ceramic ring or disk vibrating in the thickness direction.

For the sandwiched piezoelectric ultrasonic transducer form in Figure 1, it has been researched based on Mason’s model by Lin S.Y. et al. from the perspective of theory analysis [13,14,15]. But with this method it is not easy to combine with some circuits such as excitation circuits, filters and amplifiers, diodes, and so on. Also, the required parameters cannot be got easily, which primarily includes resonance and anti-resonance frequencies, the input electrical impedance and phase, input reactance, and the mass vibrating speed. Therefore, in order to solve these problems above, by means of the SPICE equivalent circuit model [10], a PSpice equivalent circuit model of the sandwiched piezoelectric ultrasonic transducer vibrating in longitudinal mode is given in this paper. Based on the proposed PSpice model, the impedance and transient analysis are performed so as to obtain the resonance and anti-resonance frequencies, the vibrating speed ratio between the front mass and the back mass. For further verification of the accuracy and application of the proposed PSpice model, a pitch-catch setup and an experimental platform are built. The analysis in time and frequency domains is carried out by the pitch-catch setup. It can be found that the simulated results have good consistency with the experimental ones.

## 2. Materials and Methods

For establishing the PSpice model of the sandwich piezoelectric ultrasonic transducer vibrating in longitudinal direction, the one-dimensional wave and transmission line theories for the piezoelectric vibrational mechanism in thickness mode are illustrated in this section.

### 2.1. Transmission Line and Wave Theories

**Figure 2:**
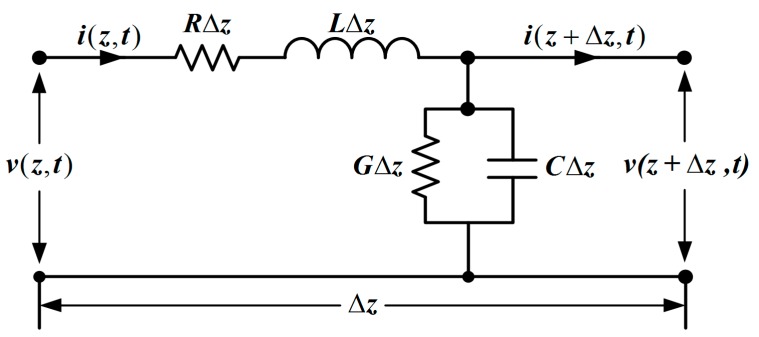
The circuit structure diagram of a transmission line of a length of Δ*z*.

To obtain the proper parameters used in simulations, the comparison of wave propagation is carried out in electrical transmission lines and acoustical medium, respectively. Consider a different length Δz of a transmission line from Figure 2, which is illustrated by the parameters as follows [16]:

- R represents the resistance per unit length in $\Omega \cdot m^{-1}$,
- L represents the inductance per unit length in $H\cdot m^{-1}$,
- G represents the conductance per unit length in $S\cdot m^{-1}$,
- C represents the capacitance per unit length in $F\cdot m^{-1}$.

Note that R and L are connected in series, G and C are connected in parallel.

To obtain the above parameters, Kirchhoff’s voltage law is used in the circuit from Figure 2, we have

(1) $$v(z,t)-R\Delta zi(z,t)-L\Delta z\frac{\partial i(z,t)}{\partial t}-v(z+\Delta z,t)=0$$

which leads to

(2) $$-\frac{v(z+\Delta z,t)-v(z,t)}{\Delta z}=Ri(z,t)+L\frac{\partial i(z,t)}{\partial t}$$

With the limit as Δz→0, Equation (2) becomes

(3) $$-\frac{\partial v(z,t)}{\partial z}=Ri(z,t)+L\frac{\partial i(z,t)}{\partial t}$$

Similarly, by using Krichhoff’s current law to the node N in Figure 2, we can derive

(4) $$i(z,t)-G\Delta zv(z,t)-C\Delta z\frac{\partial v(z+\Delta z,t)}{\partial t}-i(z+\Delta z,t)=0$$

Then letting Δz→0, we can obtain

(5) $$-\frac{\partial i(z,t)}{\partial z}=Gv(z,t)+C\frac{\partial v(z,t)}{\partial t}$$

We call Equations (3) and (5) the general transmission-line equations, which are first-order partial differential equations based on v(z,t) and i(z,t). For simplifying the pair partial differential equations, the time harmonic cosine function is used and the voltage v(z,t) and the current i(z,t) can be expressed as

(6) $$v(z,t)=real\left[V(z)e^{j\omega t}\right]$$

(7) $$i(z,t)=real\left[I(z)e^{j\omega t}\right]$$

where ω is the angular frequency.

The general transmission line equations on the basis of V(z) and I(z) can be obtained

(8) $$-\frac{dV(z)}{dz}=(R+j\omega L)I(z)$$

(9) $$-\frac{dI(z)}{dz}=(G+j\omega C)V(z)$$

Equations (8) and (9) are called as time-harmonic transmission line equations which simplify to the following Equations (10) and (11) under the lossless conditions (R=0, G=0).

(10) $$-\frac{dV(z)}{dz}=j\omega LI(z)$$

(11) $$-\frac{dI(z)}{dz}=j\omega CV(z)$$

In order to obtain the propagation constant and characteristic impedance of transmission line, the time-harmonic transmission line equations are used. By means of differentiating them with respect to z, we can obtain [16]

(12) $$\frac{d^{2}V(z)}{dz^{2}}-\gamma^{2}V(z)=0$$

(13) $$\frac{d^{2}I(z)}{dz^{2}}-\gamma^{2}I(z)=0$$

where γ is called as the propagation constant. It is composed of an attenuation constant α in Np/m and a phase constant β in rad/m. It can be expressed as

(14) $$\gamma =\alpha +j\beta =\sqrt{\left(R+j\omega L)(G+j\omega C\right)}$$

The general solution of the differential Equation (12) is denoted as

(15) $$V(x)=Ae^{-(\alpha +j\beta )z}+Be^{(\alpha +j\beta )z}$$

and Equation (13) has the same form solution.

The time dependence for Equation (15) can be got by means of multiplying $e^{j\omega t}$, we can obtain

(16) $$v(x,t)=V(x)e^{j\omega t}=Ae^{-\alpha z}e^{j(\omega t-\beta z)}+Be^{\alpha z}e^{j(\omega t+\beta z)}$$

Equation (16) illustrates two traveling waves. One travels in the positive z direction with an amplitude A and it decays at a rate α, while the other travels in the opposite direction with an amplitude B and has the same decay-rate. The propagation of an acoustical wave is controlled by a pair of differential equations which have the same type as Equations (12) and (13). In the situation of harmonic waves, corresponding with Equations (12) and (13), the linearized acoustic plane wave with lossy Equations can be obtained [17]:

(17) $$\frac{\partial^{2}p(z,t)}{\partial^{2}z}+k_{c}^{2}p(z,t)=0$$

(18) $$\frac{\partial^{2}u(z,t)}{\partial^{2}z}+k_{c}^{2}u(z,t)=0$$

here, p(z,t) is called the pressure in Pa and u(z,t) represents the particle velocity in m/s. Corresponding to γ, $k_{c}$ denotes also the complex wave number consisted of an attenuation constant α in Np/m and a wave number k in rad/m. The complex wave-number $k_{c}$ can be expressed as

(19) $$k_{c}=\frac{\omega }{v_{t}}\frac{1}{\sqrt{1+j\omega \tau }},\;v_{t}=\sqrt{E/\rho }$$

here, τ is the relaxation time and $v_{t}$ is the sound speed, E represents Young’s modulus and ρ is material density.

The general solution for Equation (17) is expressed as

(20) $$p(z,t)=Ae^{-\alpha z}e^{-j(\omega t-kz)}+Be^{\alpha z}e^{j(\omega t+kz)}$$

and is corresponding with the electrical transmission line’s solution Equation (16). In addition, Equation (18) has a solution of the same form. Combined with Equations (19) and (20), we can derive

(21) $$\alpha =\frac{\omega }{v_{t}}\frac{1}{\sqrt{2}}{\left[\frac{\sqrt{1+{\left(\omega \tau \right)}^{2}}-1}{\sqrt{1+{\left(\omega \tau \right)}^{2}}}\right]}^{\frac{1}{2}}$$

(22) $$k=\frac{\omega }{v_{t}}\frac{1}{\sqrt{2}}{\left[\frac{\sqrt{1+{\left(\omega \tau \right)}^{2}}+1}{\sqrt{1+{\left(\omega \tau \right)}^{2}}}\right]}^{\frac{1}{2}}$$

The characteristic impedance $Z_{el}$ of the lossy transmission line is given as [16]

(23) $$Z_{el}=\sqrt{\frac{R+j\omega L}{G+j\omega C}}$$

The characteristic acoustic impedance $Z_{a}$ of the lossy acoustical medium is represented as

(24) $$Z_{a}=\rho v_{t}\sqrt{1+j\omega \tau }$$

here, ρ denotes the medium’s density in $kg/m^{3}$. Equations (23) and (14) are expanded in order to approximate the characteristic impedance and propagation constant by reserving the low order parts. They are rewritten as

(25) $$Z_{el}≅\sqrt{\frac{L}{C}}\left[1+\frac{1}{2j\omega }(\frac{R}{L}-\frac{G}{C})\right]$$

(26) $$\gamma ≅\frac{1}{2}\sqrt{LC}(\frac{R}{L}+\frac{G}{C})+j\omega \sqrt{LC}$$

Now we take small but non-negligible losses into consideration and suppose R<<ωL, G<<ωC and ωτ<<1. Based on these assumptions, the second term of Equation (25) is neglected, only keeping the $\sqrt{LC}$ as the characteristic impedance. Likewise, according to Equation (24), the low acoustical characteristic impedance is derived as $\rho v_{t}$. In addition, the wave-number k to Equation (22) can be approximately expressed as $\omega /v_{t}$. By using the approximate assumptions mentioned above again, according to Equation (26) the phase constant β can be obtained as $\omega \sqrt{LC}$. For the purpose of correlating the two theories, the impedance type analogy relationship is selected in which the mechanical force is denoted by the voltage and the current denotes particle velocity. The equivalence between the systems is denoted as

(27) $$Z_{el}≅Z_{a}A=A\rho v_{t}$$

here, A is the cross-sectional area for the acoustic beam in $m^{2}$.

The relationship of the low-loss characteristic impedance Equation (27) is used to obtain the following expressions

(28) L≡Aρ

(29) $$C\equiv \frac{1}{A\rho v_{t}^{2}}$$

The real part of Equation (26) is called as the attenuation constant α as follows:

(30) $$\alpha =\frac{1}{2}\sqrt{LC}\left(\frac{R}{L}\right)+\frac{1}{2}\sqrt{LC}\left(\frac{G}{C}\right)$$

Corresponding to Equation (30), the classical theory relationship of acoustic attenuation is obtained as

(31) $$\alpha_{classical}=\alpha_{v}+\alpha_{tc}$$

here, $\alpha_{v}$ denotes the attenuation coefficient resulting from viscous losses while $\alpha_{tc}$ is the attenuation coefficient deriving from the thermal conduction.

According to Equations (28)–(30), we can derive the following expressions

(32) $$R\equiv 2\rho v_{t}A\alpha_{v}$$

(33) $$G\equiv \frac{2\alpha_{tc}}{\rho v_{t}A}$$

Because of the materials used in the sandwich piezoelectric ultrasonic transducer having a low heat conductance, the loss resulting from the thermal conductance can be neglected. Then letting the conductance G=0, we can get

(34) $$\alpha =\alpha_{v}=\frac{R}{2}\sqrt{\frac{L}{C}}=\frac{\omega }{\omega }\frac{R}{2}\frac{1}{Lv_{t}}=\frac{\omega }{2v_{t}}\tan\delta_{m}$$

here, $\tan\delta_{m}=1/Q_{m}$ is mechanical loss factor and $Q_{m}$ is mechanical quality factor.

Therefore, Equation (32) can be rewritten as

(35) $$R\equiv \omega L/Q_{m}=L\omega \tan\delta_{m}$$

Finally, the parameters of the acoustical lossy transmission line can be derived as follows:

(36) $$L=A\rho \;C=\frac{1}{A\rho v_{t}^{2}}\;R=\frac{\omega L}{Q_{m}}\;G=0$$

### 2.2. Piezoelectric Ceramic Ring Vibrating in Thickness Mode

The thickness direction vibration of the thickness poled piezoelectric ceramic rings is a general vibration mode. For this kind of thickness poled piezoelectric ceramic ring, its thickness is much lower than diameter. It is assumed that it works in the thickness mode. For the piezoelectric ring with fixed or free ends vibrating in thickness mode, it has a fundamental resonant frequency [18]:

(37) $$f=\frac{v_{t}}{2l_{0}}$$

here, $v_{t}$ denotes acoustic velocity in piezoelectric material and $l_{0}$ denotes the thickness of a piezoelectric ceramic ring.

**Figure 3:**
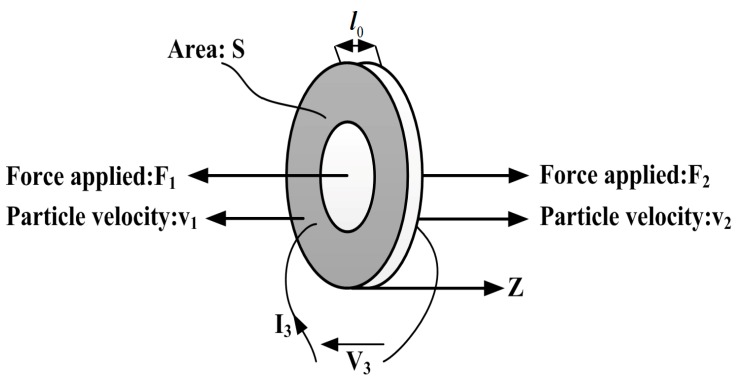
Piezoelectric ceramic ring vibrating in thickness mode.

$F_{1}$, $F_{2}$ are the external forces applied to the back and front surfaces of the piezoelectric ceramic ring, $l_{0}$ is its thickness, S indicates its cross-section area, $v_{1}$ and $v_{2}$ represent the particle speed, $V_{3}$, $I_{3}$ denotes the voltage and current respectively and the Z axis indicates the vibrating direction as shown in Figure 3.

**Figure 4:**
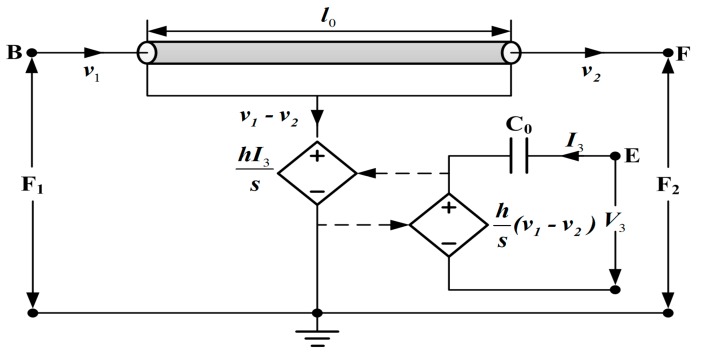
Leach’s equivalent circuit model for a thickness vibration piezoelectric ring.

For modeling a sandwiched piezoelectric ceramic transducer vibrating in longitudinal mode, the piezoelectric ring is modeled using Leach’s equivalent circuit model [9] as shown in Figure 4.

It consists of the clamped capacitance $C_{0}$, a transmission line used to represent the mechanical parts of the piezoelectric ring, and two controlled sources used for indicating the coupling between the electrical and mechanical sections of the piezoelectric ring. Here, $h_{33}$ is the piezoelectric constant, s is the Laplace operator, the nodes B, E and F represent the back face, the front face and the electrical terminal of the piezoelectric ceramic ring, respectively. For the electrical part, the clamped capacitance $C_{0}$ is given as follows

(38) $$C_{0}=\frac{A}{\beta_{33}^{S}l_{0}}$$

here, $\beta_{33}^{S}$ is the clamped dielectric impermeability, the cross-section area $A=\pi (R_{0}^{2}-R_{i}^{2})$.

## 3. PSpice Modeling

For establishing the PSpice model of the sandwiched piezoelectric ceramic ultrasonic transducer vibrating in longitudinal direction, the piezoelectric ceramic stack from Figure 1 is modeled using Leach’s equivalent circuit model from Figure 4, and non-piezoelectric elements including the front and back masses, and metal electrodes from Figure 1 are modeled by using the lossless transmission line as shown below.

### 3.1. Modeling of Non-Piezoelectric Elements

**Figure 5:**
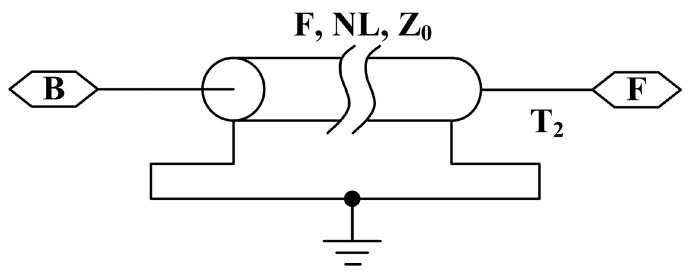
The lossless transmission line model in PSpice.

For simplifying the ultrasonic transducer model, the metal front and back masses and metal electrodes are modeled by using the lossless transmission line model from Figure 5.

In the simulation using PSpice, the parameters of the lossless transmission line mainly include the resonance frequency F, the normalized length of the transmission line NL and the characteristic impedance $Z_{0}$. Then, their expressions are given as [19]:

(39) $$F=\frac{NL}{LEN}v_{t}$$

(40) $$Z_{0}=\rho v_{t}S$$

here, $v_{t}$ is the material sound velocity, S represents the cross-sectional area and LEN denotes the length of the transmission line.

### 3.2. Modeling of the Piezoelectric Ceramic Stack Vibrating in Longitudinal Direction

**Figure 6:**
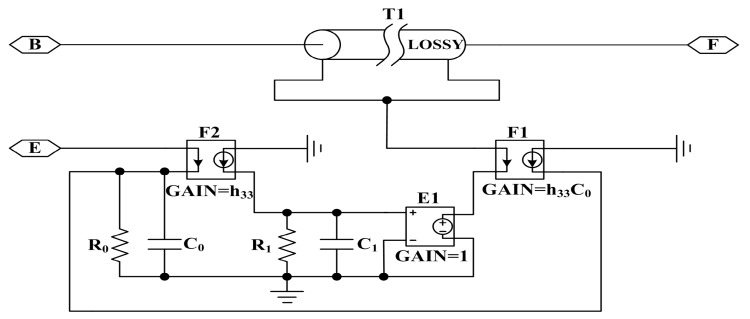
PSpice circuit of the Leach’s model of a piezoelectric ceramic ring vibrating in thickness direction.

The single piezoelectric ceramic ring is modeled using the PSpice model from Figure 6, and the model takes losses including mechanical and dielectric losses into consideration by means of the lossy transmission line and resistance $R_{0}$ used to represent dielectric loss.

The parameters of the lossy transmission line are obtained from Equation (36), and the loss resistance $R_{0}$ is given as [20]

(41) $$R_{0}=\frac{1}{C_{0}\tan\delta_{e}\omega }$$

here, $\tan\delta_{e}=1/Q_{e}$ is called as the dielectric loss factor and $Q_{e}$ is electrical quality factor. Then, resistance [10] $R_{1}$ has the value $R_{1}=1\;k\Omega$ and capacitance $C_{1}$ has the value $C_{1}=1\;\mu F$.

**Figure 7:**
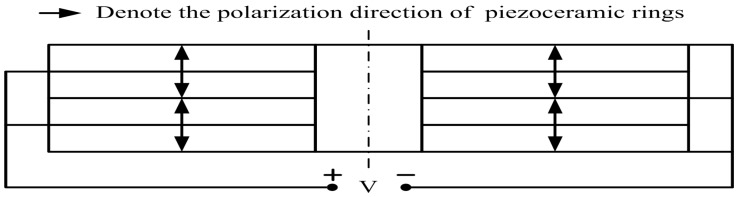
The piezoelectric ceramic stack composed of four same thickness-poled rings.

In Figure 7, V denotes the voltage. The piezoelectric ceramic stack is composed of four same poled rings and four metal electrodes as shown in Figure 7. Therefore we need four PSpice models of the piezoelectric rings from Figure 6 and four lossless transmission line models from Figure 5 to implement the model of a piezoelectric ceramic stack.

**Figure 8:**
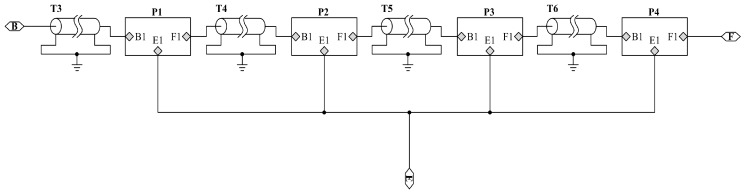
PSpice circuit model of a piezoelectric ceramic stack with electrodes.

The piezoelectric rings located in the piezoelectric ceramic stack are connected together mechanically in series as shown in Figure 8. However, they belong to parallel relationship in the electrical terminals [21]. Here, T3, T4, T5, T6 are the metal electrodes and P1, P2 and P3, P4 are the piezoelectric ceramic rings.

### 3.3. The Sandwich Piezoelectric Ultrasonic Transducer Model with PSpice

**Figure 9:**
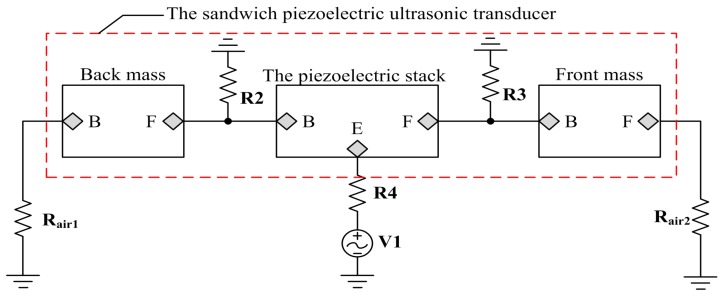
The AC analysis simulation circuit of the sandwiched piezoelectric ultrasonic transducer.

On the basis of the above analysis about the modeling of the piezoelectric stack and the metal masses, we can obtain the PSpice model of the sandwich piezoelectric ultrasonic transducer from Figure 9.

In Figure 9, the resistances $R_{air1}$ and $R_{air2}$ are used to model air load and have the value [22] $R_{air1}=R_{air2}=0.0263\;\Omega$. Also, the resistances R2 and R3 are used to represent the bonded layer and have the value R2=R3=1 MΩ. Then the resistance R4 used to model the internal resistance of AC voltage source $V_{1}$ has the value R4=50 Ω.

The sandwich piezoelectric ceramic ultrasonic transducer is mentioned in this paper and the piezoelectric material selects PZT-4 [23], and the metal materials [24] used in the front and back masses are hard aluminum and steel, respectively. The detailed parameters of these materials are given in Table 1, Table 2, Table 3 and Table 4. Here, it should be pointed that $l_{1}$ is the length of the back mass, $l_{2}$ is the length of the front mass and $l_{3}$ is the thickness of a metal electrode ring. For the purpose of verification the accuracy of the proposed PSpice model of the piezoelectric ultrasonic transducer, the impedance analysis based on the simulation circuit from Figure 9 is carried out as shown in below.

### 3.4. Impedance Analysis

**Figure 10:**
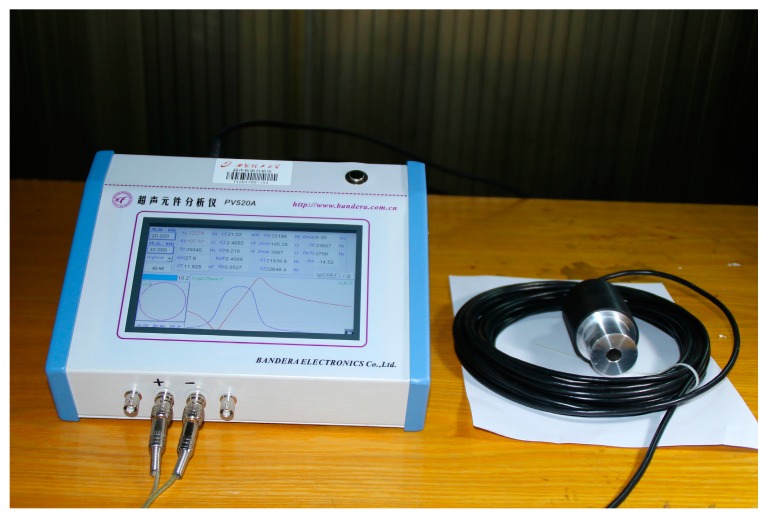
The impedance test of the sandwiched ultrasonic transducer.

**Figure 11:**
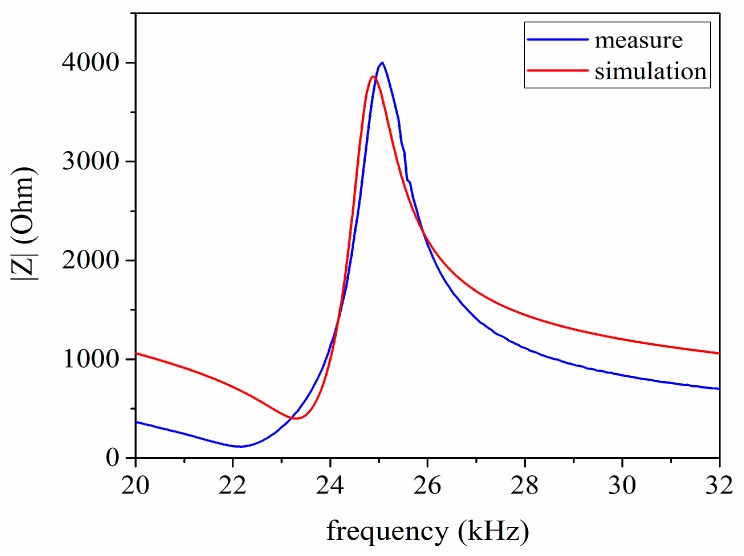
The impedance-frequency relationship of the sandwiched ultrasonic transducer.

The impedance analysis is used to research the frequency response and derive the resonance and antiresonance frequencies. Moreover, the measured impedance results are obtained by utilizing the impedance analyzer PV520A which is made by BEIJING BAND EAR CO (Beijing, China), LTD as shown in Figure 10. The impedance analysis results of the sandwich ultrasonic transducer are shown in Figure 11. The resonance and anti-resonance frequencies of the measured and simulated are specially shown in Table 5. From Table 5, $f_{t}$ and $f_{m}$ represent the simulated and measured frequencies of a sandwich piezoelectric transducer, respectively and $\Delta =|f_{t}‒f_{m}|/f_{m}$. Also, $f_{s}$ and $f_{p}$ represent resonance and anti-resonance frequencies, respectively. From Table 5, it can be found that the measured results have good consistency with the simulated results.

There are several factors that can well explain frequency error or difference in frequencies in Table 5. Firstly, the standard physical parameters of the piezoelectric ceramic rings, the metal masses and the metal electrodes are applied in simulation; to a certain extent, they are different from the truthful physical parameters. Secondly, it must be met in the theoretical analysis condition that the length of the sandwiched ultrasonic transducer have to be much more than its diameter, so that the vibration of the sandwiched transducer can be approximated as the longitudinal vibration of an extended composite round bar. However, it is not possible in practical cases. Thirdly, based on the above analysis, the prestressed bolt and the epoxy resin used for sealing the sandwiched transducer are ignored in the proposed PSpice model. Here, it should be pointed out that the prestressed bolt has the effect on the electrical impedance with resulting in some frequency error. In addition, the sealing epoxy resin can lead to some frequency error for it changing the vibrator length. However, for the practical transducers, these will not be neglected.

### 3.5. Transient Analysis

**Figure 12:**
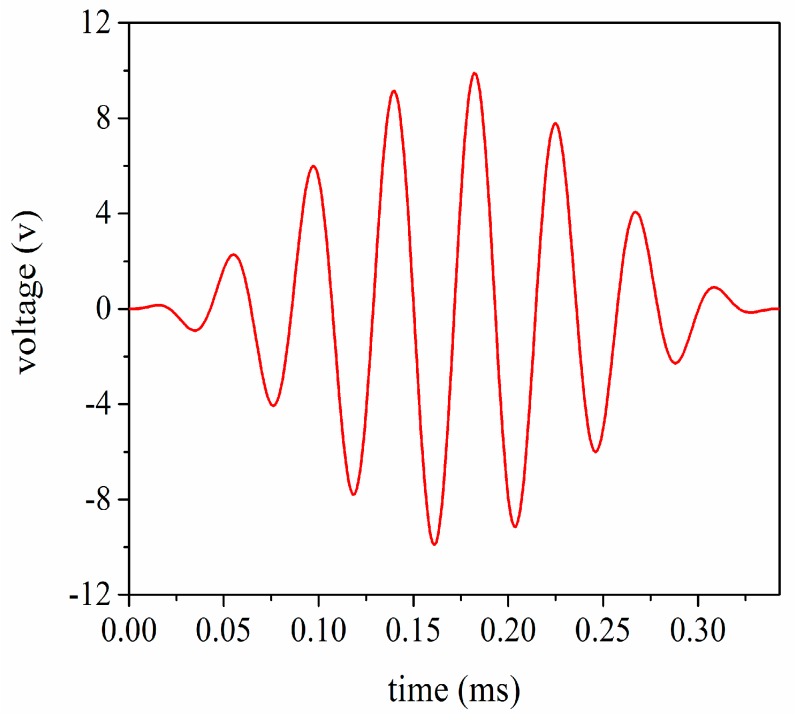
The single tone signal modulated by Hanning window.

In order to investigate the vibrational velocity ratio between the front and back masses, the transient analysis is carried out using the 8 cycle single tone signal by modulated Hanning window as shown in Figure 12. At the same time, in order to obtain the large vibration speed in the front mass, the front and back masses chose heavy metals and light metals, respectively. According to the momentum conservation law, we can get

(42) $$\begin{array}{l} m_{F}v_{F}=m_{B}v_{B}\\ \frac{v_{F}}{v_{B}}=\frac{m_{B}}{m_{F}} \end{array}$$

**Figure 13:**
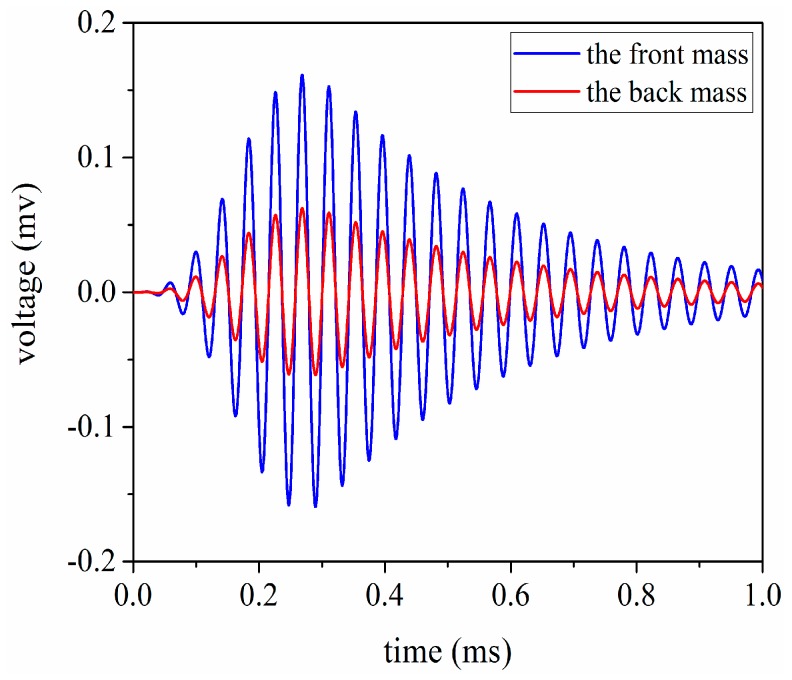
The vibrational velocity ratio between the front and back masses.

In general, if the materials of the front and back masses are chosen as aluminum and steel respectively, the vibrational velocity ratio between the front mass and the back mass is 3:1 for sandwich piezoelectric ultrasonic transducers. From Figure 13, the simulation result for the vibrational velocity ratio is obtained as

(43) $$\frac{v_{F}}{v_{B}}=\frac{0.32}{0.12}\approx 2.7:1$$

here, $v_{F}$ and $v_{B}$ represent the vibrational velocities of the front and the back masses, respectively.

## 4. The Pitch-Catch Setup

**Figure 14:**
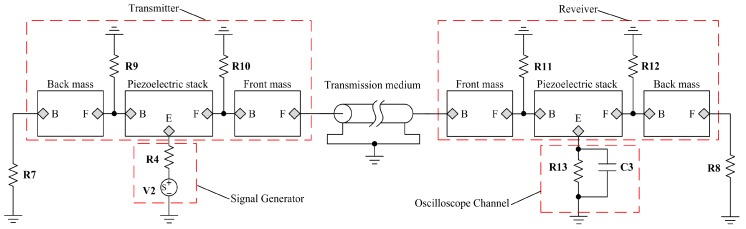
The simulation circuit of the pitch-catch setup.

To demonstrate the application of the proposed model, a pitch-catch setup is built as shown in Figure 14. On the basis of the setup, AC and transient analysis are carried out. The simulations are compared with measurements in the time domain. It primarily includes one transmitter used to produce an ultrasonic wave signal, one receiver used to receive the ultrasonic wave signal and a piece of steel plate used as a propagation medium.

Here, the resistance R4 has the value R4=50 Ω, the resistance R13 has the value R13=10 MΩ and the capacitance C3 has the value C3=3.9 pF. They are used to model the input impedance and capacitance of the recording channel of an oscilloscope. Also these resistances R9, R10 and R11, R12 are used to represent bonded layers and have the value R9=R10=R11=R12=1 MΩ. Then the resistances R7 and R8 are used to model air load and have the value R7=R8=0.0263 Ω.

**Figure 15:**
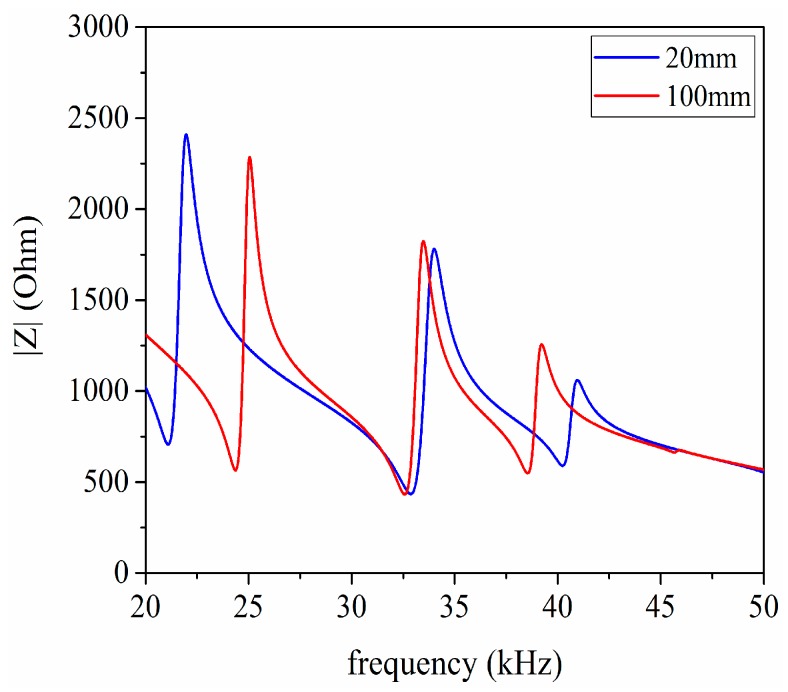
The impedance analysis of the pitch-catch setup.

In the first place, for the purpose of obtaining the resonance frequencies of the pitch-catch setup, the AC analysis is performed under the conditions of the length of the transmission medium having the value 20 mm and 100 mm. But the excitation source V2 from Figure 14 need to be substituted by the AC voltage source which is the same as V1. The impedance analysis result is given as shown in Figure 15. According to Figure 15, it can be found that the first resonance frequencies are 21.098 and 24.363 in kHz, respectively.

In the next place, based on the pitch-catch setup from Figure 14, the transient analysis is carried out and the single tone signal modulated by Hanning window is chosen as excitation signal. The detailed analysis scheme is shown as follows:

① the length of the transmission medium is 20 mm and the excitation signal frequencies are 23.309 and 21.098 in kHz.
② the length of the transmission medium is 100 mm and the excitation signal frequencies are 23.309 and 24.363 in kHz.

**Figure 16:**
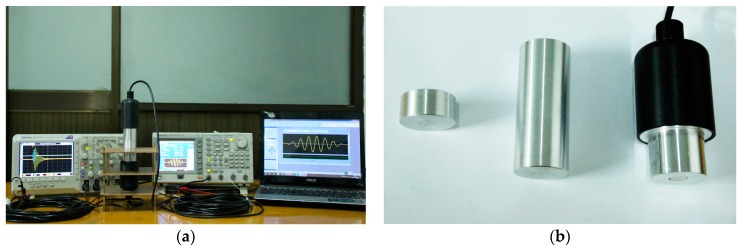
The pitch-catch experimental platform, (**a**) experimental testing; (**b**) Aluminum cylinders and sandwiched piezoelectric ultrasonic transducers.

Then, to verify the accuracy of the simulated results, the pitch-catch experimental platform is built from Figure 16, which includes the arbitrary/functional generator used to generate the excitation signal, oscilloscope used to record the voltage signal and sandwich ultrasonic transducers used to transmitting and receiving the ultrasonic wave signal, the aluminum rods used to transmit the ultrasonic wave signal. For the transient analysis of the pitch-catch setup, the single tone signal modulated by Hanning window is selected as the excitation signal. Moreover, in order to make the transducer and the transmission medium fit tightly, the glycerin is used as couplant.

**Figure 17:**
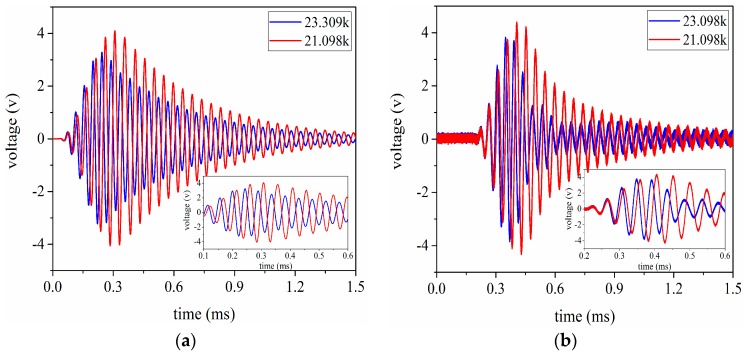
The signal waveform received by the ultrasonic transducer under the condition that the aluminum cylinder is 20 mm in length, (**a**) the simulated results; (**b**) experimental results.

**Figure 18:**
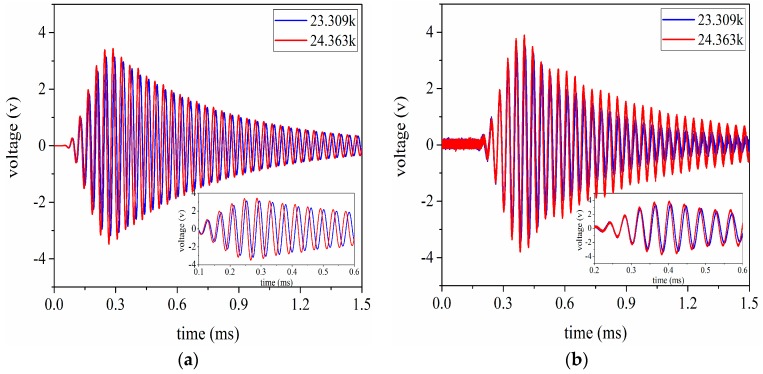
The signal waveform received by the ultrasonic transducer under the condition that the aluminum cylinder is 100 mm in length, (**a**) the simulated results; (**b**) experimental results.

The voltage signals are received by the ultrasonic transducer under the conditions that the aluminum cylinders are 20 mm and 100 mm in length respectively. Simultaneously, these signal data are recorded by the oscilloscope. By using these recorded data, the signal waveforms received in the time domain are given as shown in Figure 17 and Figure 18. According to Figure 17 and Figure 18, it can be easily found that the simulated results have good consistency with the experimental ones. In addition, the conclusion can also be drawn that the optimal excitation frequency for the pitch-catch setup is not necessarily the resonance frequency for the sandwiched ultrasonic transducer, because the resonance frequency can be obtained under the condition of no load.

At the same time, the voltage values received are specifically listed in Table 6 and Table 7 under the length of the transmission medium having 20 mm and 100 mm respectively. From Table 6 and Table 7, $V_{s1}$ and $V_{s2}$, $V_{m1}$ and *V_m*2*_*. represent the simulated and measured voltage values from the received ultrasonic transducer respectively and $\Delta_{1}=|V_{s1}‒V_{m1}|/V_{m1}$, $\Delta_{2}=|V_{s2}‒V_{m2}|/V_{m2}$.

There are several factors that can well explain amplitude error or difference in the received voltage in Table 6 and Table 7. Firstly, it is impossible to keep the same for each manufactured transducer. Secondly, the standard physical parameters of the transmission medium are applied in the simulation calculation, which may be different from the truthful physical parameters. Thirdly, due to the effect of frequency error and prestressed bolt for this transducer, this also can lead to relatively large amplitude error.

## 5. Discussion

The PSpice model of the sandwiched piezoelectric ceramic ultrasonic transducer in longitudinal vibration is proposed in this work. It is mainly for providing convenience for the design and analysis of the sandwiched ultrasonic transducers. Compared with the theory analysis method based on the Mason’s equivalent circuit of the sandwiched piezoelectric ultrasonic transducers, the proposed PSpice model has the following advantages. First, it is very easy to access the parameters such as the input electrical impedance and reactance, the vibrational velocity, the resonance and anti-resonance frequencies. Second, the proposed transducer model has great flexibility and strong expansion. Moreover, it can easily combine with the excitation, filter and amplifier circuits, which provides help to improve and optimize these circuits. Third, on the basis of the model proposed, the wireless power or data transmission system based on the sandwiched ultrasonic transducer in longitudinal or thickness vibration can be easily built; the parameters for the system are easily obtained by the AC and transient analysis, and so on.

The effect of the losses including mechanical and dielectric on the transducer performance parameters such as resonance frequency, electrical quality factor and electro-acoustical efficiency, needs be considered in practical transducers. However, these mechanical losses, such as the metal front and back masses, and the metal electrodes, are ignored in the proposed PSpice model. Therefore, for the purpose of improving the sandwiched piezoelectric ceramic transducer in longitudinal or thickness vibration, the effect of the losses for the transducer performance should be analyzed by using the lossy transmission line. Moreover, the effect of the sealed epoxy resin layer is not considered in the transducer PSpice model. For the formulation of the sealed epoxy resin layer, it can be regarded as the lossy transmission line. It is strongly suggested that the effect of the prestressed bolt can be analyzed using the finite element method. These issues are expected to be further investigated in our subsequent work.

## 6. Conclusions

In this paper, based on Leach’s equivalent circuit and lossless transmission line, a PSpice model of the sandwiched piezoelectric ultrasonic transducer in longitudinal vibration is proposed, and the resonance and antiresonance frequencies are obtained. To further verify the accuracy and application of the proposed model, a pitch-catch setup and an experimental platform are built; the resonance frequency is obtained and the simulated results are compared with the measured ones. In summary, based on the analysis mentioned above, some conclusions can be drawn as follows:

(1) The comparisons of the measured results and simulated values of the sandwiched piezoelectric ultrasonic transducer indicate the accuracy of the proposed lossy model.
(2) The PSpice model has been successfully applied to the pitch-catch setup. It is shown that the experimental results and the simulated values have good consistency. Simultaneously, we can find that the optimal excitation frequency of the sandwiched ultrasonic transducer is not necessarily the resonance frequency for the pitch-catch setup.
(3) The accomplishment in PSpice can provide convenient analysis for the sandwiched piezoelectric transducers in time and frequency domains. Compared with the sandwiched piezoelectric transducer model based on Mason’s equivalent circuit, the proposed model may be more easily used to investigate the sandwiched transducers.
(4) The proposed PSpice model of sandwich piezoelectric transducers can be more conveniently used to combine with other circuits such as driving circuits, filters, amplifiers, and so on.

## Figures and Tables

**Table 1:** The material parameters of the piezoelectric ceramic PZT-4.

Parameters	Value
$\rho_{1}$ (kg/m^3^)	7500
$v_{t}$ (m/s)	4600
*A* (m^2^)	957.4 × 10^−6^
$l_{0}$ (m)	5 × 10^−3^
$Q_{m}$	500
$\tan\delta_{e}$	0.004
$\beta_{33}^{S}$ (m/F)	1.78 × 10^8^
$h_{33}$ (V/m)	27.12 × 10^8^

**Table 2:** The material parameters of the aluminum used in the front mass.

Parameters	Value
$\rho_{2}$ (kg/m^3^)	2700
$v_{t}$ (m/s)	5037
$S_{2}$ (m^2^)	1075.2 × 10^−6^
$l_{2}$ (m)	43 × 10^−3^

**Table 3:** The material parameters of the steel used in the back mass.

Parameters	Value
$\rho_{3}$ (kg/m^3^)	7800
$v_{t}$ (m/s)	5262
$S_{1}$ (m^2^)	1134.1 × 10^−6^
$l_{1}$ (m)	45 × 10^−3^

**Table 4:** The material parameters of the copper used in the metal electrodes.

Parameters	Value
$\rho_{4}$ (kg/m^3^)	8900
$v_{t}$ (m/s)	3718
$S_{1}$ (m^2^)	1134.1 × 10^−6^
$l_{3}$ (m)	0.5 × 10^−3^

**Table 5:** The measured and simulated resonance and anti-resonance frequencies for a sandwich piezoelectric ultrasonic transducer.

Parameters	$f_{t}$	$f_{m}$	Δ%
$f_{s}(kHz)$	23.309	22.2	4.99%
$f_{p}(kHz)$	24.885	25.1	0.86%

**Table 6:** The measured and simulated voltage values under the condition of the length for the transmission medium having 20 mm.

Frequency (kHz)	*V_S_*_1_(*V*)	*V_m_*_1_(*V*)	Δ_1_%
23.309	6.65	7.76	14.3%
21.098	8.16	8.88	8.1%

**Table 7:** The measured and simulated voltage values under the condition of the length for the transmission medium having 100 mm.

Frequency (kHz)	*V_S_*_2_(*V*)	*V_m_*_2_(*V*)	Δ_2_%
23.309	6.32	6.96	9.2%
24.363	6.92	7.76	10.8%
